# Efficacy of pembrolizumab rechallenge for metastatic urothelial carcinoma

**DOI:** 10.1002/iju5.12474

**Published:** 2022-05-25

**Authors:** Nobutaka Nishimura, Makito Miyake, Takuto Shimizu, Akira Tachibana, Nobumichi Tanaka, Kiyohide Fujimoto

**Affiliations:** ^1^ Department of Urology Nara Medical University Kashihara Nara Japan; ^2^ Department of Urology Saiseikai Chuwa Hospital Sakurai Nara Japan; ^3^ Department of Urology Gokeikai Osaka Kansei Hospital Yodogawaku Osaka Japan

**Keywords:** immune checkpoint inhibitor, metastatic urothelial cancer, pembrolizumab rechallenge

## Abstract

**Introduction:**

Before the approval of enfortumab vedotin, no standard treatment was available as the salvage treatment for patients with metastatic urothelial carcinoma who had failed second‐line or later pembrolizumab. Pembrolizumab rechallenge is one of the options for these patients, but there is limited evidence on the effectiveness of pembrolizumab rechallenge. We report three patients with metastatic urothelial carcinoma treated with pembrolizumab rechallenge and discuss rechallenge of immune checkpoint inhibitors in other malignancies.

**Case presentation:**

This study included three cases treated with pembrolizumab rechallenge with the age of 54 (Case 1), 78 (Case 2), and 67 (Case 3) years old. A complete response to the prior pembrolizumab was observed only in Case 1. However, no patients responded to the pembrolizumab rechallenge.

**Conclusion:**

Pembrolizumab rechallenge is not recommended in the current clinical setting of metastatic urothelial carcinoma, even for patients who showed complete response to the prior pembrolizumab.

**Abbreviations & Acronyms**
CR: complete respopnse
CT: computed tomography
GC: gemcitabine plus cisplatin
GCarbo: gemcitabine plus carboplatin
ICI: immune checkpoint inhibitor
mUC: metastatic urothelial carcinoma
OS: overall survival
PD: progression disease
PFS: progression‐free survival
PR: partial response
SD: stable disease

**Keynote message**

The effectiveness of pembrolizumab rechallenge is limited after progression disease of prior pembrolizumab.

## Introduction

In recent years, treatment methods for mUC have advanced. The standard first‐line treatment for patients with mUC has been platinum‐based chemotherapy.^1^ In December 2017 in Japan, pembrolizumab, an ICI, was approved to treat mUC that relapsed or progressed after first‐line platinum‐based chemotherapy.^2^ Moreover, in September 2021, enfortumab vedotin was approved by Japanese health insurance as the salvage treatment for patients who failed both chemotherapy and pembrolizumab.^3^ Before this, no standard treatment was available as the salvage treatment for patients with mUC who failed second‐line of pembrolizumab. The taxane‐based chemotherapy was one of the treatment options; otherwise, pembrolizumab rechallenge was performed in selected patients.^4^

The rechallenge of ICIs is one of the options for patients who had some response to the prior ICIs. If the patient responds to prior ICIs therapy, pembrolizumab rechallenges after the third‐line chemotherapy may provide benefit in terms of immunogenic cell death. However, there is limited evidence on the effectiveness of ICI rechallenge in advanced malignancies, including mUC. The reasons for ICI rechallenge are re‐administration after discontinuation due to adverse effects and after PD. This report describes three patients who developed PD after the prior pembrolizumab and were treated later with pembrolizumab rechallenge.

## Case presentation

### Case 1

**Fig. 1:**
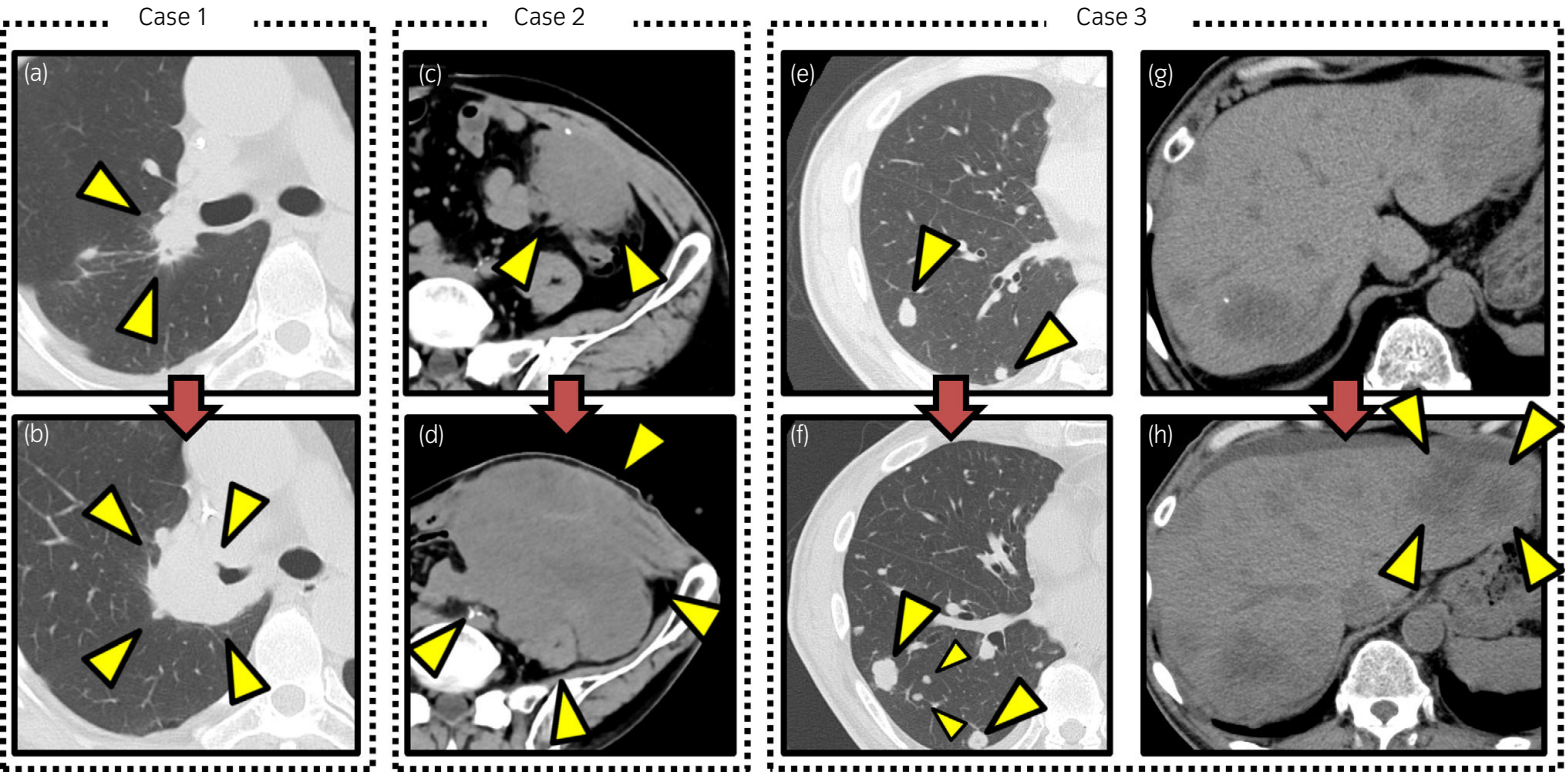
CT images before and after pembrolizumab rechallenge. (a, b) CT images in Case 1. The middle lobe metastasis in the right lung developed before pembrolizumab rechallenge (a). After five doses, the lung metastasis progressed, and the right main bronchus was compressed (b). (c, d) CT images in Case 2. Peritoneal metastasis developed before the pembrolizumab rechallenge. PD was observed immediately after three doses of pembrolizumab rechallenge (d). (e, h) CT images in Case 3. New multiple lungs and liver metastases recurred before the pembrolizumab rechallenge (e, f). After two doses of pembrolizumab rechallenge, rapid progression was observed (g, h).

The patient was a 54‐year‐old male with mUC of the left renal pelvis (cT3N0M1). He received three doses of GC. After a complete response to GC, he underwent surgical resection of the primary site. Two months later, the upper lobe metastasis in the left lung recurred, and pembrolizumab was introduced. After four doses, the lung metastasis had radiographically disappeared. A total of 13 doses were administered, and then the middle lobe metastasis in the right lung developed. After seven doses of re‐administered GC, the metastasis of the right lung progressed, and cisplatin‐induced hearing impairment was observed. Pembrolizumab rechallenge was performed (Fig. 1a). Even after the administration of five doses, the lung metastasis continued progressing over time. A respiratory failure occurred due to compression of the main bronchus (Fig. 1b), leading to the best supportive care.

### Case 2

The patient was a 78‐year‐old male with locally advanced UC of the bladder (cT3N0M0). He underwent radical cystectomy (pT4bN0M0) and three courses of adjuvant GC. Nine months later, he was found to have peritoneal metastasis, and pembrolizumab was introduced. A total of four doses were administered, but the peritoneal metastasis progressed over time. Therefore, GC was re‐administered. After three GC doses, the peritoneal metastasis had shrunk, and a partial response was observed, the peritoneal metastasis progressed, and a pembrolizumab rechallenge was performed (Fig. 1c). However, PD was observed immediately after the administration of pembrolizumab (Fig. 1d). He is alive, and pembrolizumab treatment is still ongoing.

### Case 3

The patient was a 67‐year‐old male with advanced UC of the renal pelvis (cT4N1M0). He underwent surgical resection of the primary site and subsequently received adjuvant GCarbo. After three GCarbo doses, pembrolizumab was administered due to the presence of lung and liver metastases. Multiple metastases progressed over time after a total of six doses. GCarbo was readministered, but after three doses, new multiple lung and liver metastases recurred. GCarbo was discontinued, and pembrolizumab rechallenge was performed (Fig. 1e,g). However, after two doses, rapid progression of these multiple metastases was observed, leading to the best supportive care (Fig. 1f,h).

## Discussion

**Fig. 2:**
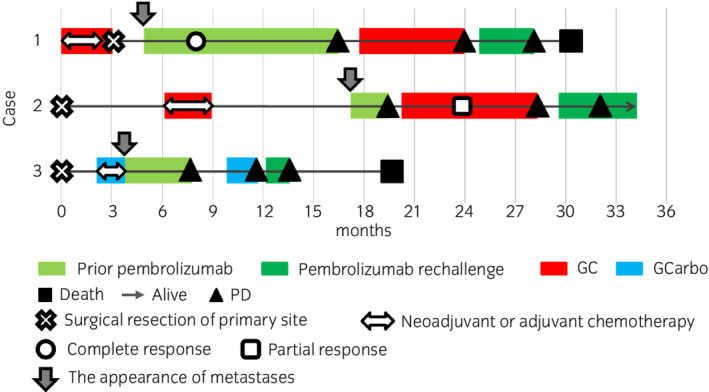
Swimmer plots describing the clinical courses. In Case 1, complete response was observed after four doses of the prior pembrolizumab. A total of 13 doses were administered, and PD was observed. However, after the pembrolizumab rechallenge, PD was immediately observed, and this patient died 4 months after the introduction of the pembrolizumab rechallenge. In Case 2, PD was observed immediately after the prior pembrolizumab and pembrolizumab rechallenge. However, pembrolizumab is still ongoing. In Case 3, PD was observed after the prior pembrolizumab and pembrolizumab rechallenge. Moreover, after pembrolizumab rechallenge, rapid progression of multiple metastases was observed. This patient died 7 months after the introduction of the pembrolizumab rechallenge.

We described the clinical courses of pembrolizumab rechallenge of three patients with mUC. Figure 2 describes the swimmer plot of the clinical courses of three patients from the prior pembrolizumab. Moreover, Table 1 summarizes each clinicopathological characteristic and clinical course. Complete response was observed only in Case 1, but the objective response to the pembrolizumab rechallenge was PD. Similar to Case 1, clinical benefit from pembrolizumab rechallenge was limited in Cases 2 and 3.

**Table 1:** Clinicopathological characteristics and clinical course of three patients receiving pembrolizumab rechallenge

Patients	OS (months)	Age (years)	Sex	Primary site	Metastatic lesions	Prior pembrolizumab		Salvage chemotherapy			Pembrolizumab rechallenge	
						Best response	PFS (months)	Regimen	Best response	PFS (months)	Best response	PFS (months)
1	23	54	Male	Renal pelvis	Lung	CR	11	GC	SD	7	PD	3
2	15	78	Male	Bladder	Peritoneum	PD	3	GC	PR	9	PD	4
3	15	67	Male	Renal pelvis	Lung, Liver	PD	4	GCarbo	PD	2	PD	2

The efficacy of pembrolizumab in the Japanese population with mUC has been demonstrated.^5^, ^6^ However, the usefulness of pembrolizumab rechallenge is still controversial. Niki *et al*. reported the effect of the ICI rechallenge after PD of first ICI in non‐small cell lung cancer.^7^ In UC, some previous reports described that ICI rechallenge had benefit when first ICI was discontinued due to the immune‐related adverse events,^8^ or when first ICI was discontinued during the response to treatment and then resumed after a drug holiday with disease progression.^9^ On the contrary, once PD is observed during the administration of ICI, ICI rechallenge might make no response. These results might show that the pembrolizumab rechallenge for UC was almost ineffective in patients with PD of the prior pembrolizumab. The number of cases is too small; therefore, further large‐scale cohort studies are required to conclude.

It might have benefit to administer different types of ICI instead of the same ICI rechallenge. Kitagawa *et al*. described that switching administrations of ICIs were effective for patients with PD of first‐line ICI in non‐small cell lung cancer.^10^ These reports have suggested that the administration of different types of ICI might improve the prognosis. In February 2021, maintenance avelumab was approved for UC that did not progress after first‐line chemotherapy.^11^ Two types of ICIs (anti‐programmed cell death protein 1 and anti programmed death‐ligand 1) are currently available in Japan in January 2022. It will be important to examine the usefulness of the sequence of these ICIs, such as salvage pembrolizumab after maintenance avelumab.

## Conclusion

We experienced three cases with mUC treated with pembrolizumab rechallenge after PD of the prior pembrolizumab. Pembrolizumab rechallenge might not be effective in the current clinical setting of mUC, even for the patient who showed complete response to the prior pembrolizumab.

## Author contributions

Nobutaka Nishimura: Conceptualization; data curation; formal analysis. Takuto Shimizu: Data curation; investigation; supervision. Akira Tachibana: Data curation. Nobumichi Tanaka: Supervision. Kiyohide Fujimoto: Conceptualization; supervision.

## Conflict of interest

The authors declare no conflict of interest.

## Approval of the research protocol by an Institutional Reviewer Board

The institutional review board of the Nara Medical University approved this study. The reference number is NMU‐2891.

## Informed consent

The opt‐out method was applied to obtain consent from participants via posters and/or websites.

## Registry and the Registration No. of the study/trial

N/A.
